# Determinants of Quality of Life in Patients With Urinary Incontinence: An Assessment Using the Incontinence Quality of Life (I-QOL) Scale

**DOI:** 10.7759/cureus.76673

**Published:** 2024-12-31

**Authors:** Hajar Mahfoudi, Ibtissam El Harch, Moncef Maiouak, Soumaya Benmaamar, Hind Bourkhime, Mohammed Omari, Noura Qarmiche, Nabil Tachfouti, Nada Otmani, Samira El Fakir

**Affiliations:** 1 Department of Epidemiology, Clinical Research and Community Health, Faculty of Medicine, Pharmacy and Dentistry of Fez, Sidi Mohamed Ben Abdellah University, Fez, MAR; 2 Biostatistics-Informatics Unit, Department of Epidemiology, Clinical Research and Community Health, Faculty of Medicine, Pharmacy and Dentistry of Fez, Sidi Mohamed Ben Abdellah University, Fez, MAR

**Keywords:** determinants, health problem, i-qol, quality of life, urinary incontinence

## Abstract

Background

Urinary incontinence is a significant health problem with physical, social, economic, and psychological consequences for patients and their quality of life. The aim of our study is to determine the impact of urinary incontinence on the quality of life and to identify its determinants in patients with this condition.

Materials and methods

A cross-sectional study was carried out in the diagnostic center of Centre Hospitalier Universitaire (CHU) Hassan II in Fez, Morocco, between June and September 2019. We used the Moroccan Arabic dialect version of the "I-QoL" (incontinence quality of life) questionnaire to assess the quality of life in patients suffering from UI.

Results

Two hundred and seventy-eight patients with urinary incontinence participated in the study. Seventy-eight percent were women, and 21.2% were men. Three-quarters (70.9%) of participants were over 50 years old. The scores for the different scales ranged from 39.05 to 54.02. "The Social Embarrassment" scale obtained the lowest score (39.05). The "Psychosocial Impact" scale had a mean score of 54.02. The total I-QoL score was 46.08 ± 24.80. Factors significantly associated with a better quality of life-related to UI (high total I-QoL score) were lighter UI: β = 11.211; 95% CI: 1.748; 20.674. Daily frequency of urination between 1 to 4 times per day: β = 8.062; 95% CI: 1.471; 14.653. Not needing to use protection: β = 9.466; 95% CI: 4.987; 13.946 or changing underwear frequently: β = 24.871; 95% CI: 19.975; 29.767. Factors significantly associated with a lower quality of life (low total I-QoL score) were rural residents (β = -8.094; 95% CI: -13.043; -3.145) after adjusting for all variables.

Conclusion

Due to Morocco's cultural diversity, which is primarily rooted in modesty, the impact of urinary incontinence on quality of life is often underestimated. This highlights the need to incorporate this aspect into treatment follow-up and guidance.

## Introduction

Urinary incontinence is defined by the International Continence Society (ICS) as any involuntary leakage of urine [1]. It is classified into stress UI (involuntary loss of urine during exertion or physical activity), urgency UI (involuntary loss of urine associated with a sudden urge to urinate), and mixed UI (urine loss associated with both urgency and exertion) [2].

UI is stigmatized in many populations, making it challenging to obtain consistent epidemiological data and explain the low rate of health-care use [2]. The prevalence of urinary incontinence is defined as the probability of being incontinent in a given population within a specific time period. It provides a static dimension of the scale of the problem. There is significant variation in reported figures in the literature, ranging from 10% to 58.4%, sometimes even higher [3]. The prevalence varies according to the type of UI, with stress incontinence accounting for 16% of cases, urgency incontinence about 19%, and mixed type over 64% [4].

UI affects women more than men. In Brazil, approximately 30% to 43% of women suffer from involuntary urinary leaks at some point in their lives; however, these rates may not reflect reality, as this condition is underdiagnosed and not well addressed by healthcare services [5].

In a study conducted in Morocco on 1,000 surveyed women, 271 reported experiencing at least one episode of incontinence in the previous month, resulting in a prevalence of 27.1%. The majority of these incontinent women (48.70%) were between 30 and 60 years old [6].

In another study of 189 women conducted in the primary health-care facilities in Fez, Morocco, during 2019, about 32.8% of the women reported that they had involuntary loss of urine; their mean age was 47.79 years (SD = 12.88 years) [4].

Male UI is rarely an isolated symptom and is most often associated with other lower urinary tract symptoms. Its prevalence tends to increase and varies between 1% and 39% depending on the definition and the studied population. The prevalence of male UI following surgical intervention also varies [7].

The costs generated by this urinary symptom are high, estimated at 2% of the health budget in European and North American countries. The perspectives suggest a significant increase due to the aging population. This knowledge should help assess the economic burden of incontinence on both a social and individual level and better understand its psychological and physical impact. Increased understanding leads to the ability to limit its consequences through modern and adapted treatments [3].

Urinary incontinence is quite frequent, and annoying symptoms can occur at any age in both men and women as well as in children. Individuals affected by it often deny and hide their urinary incontinence, leading to physical and psychosocial restrictions. The consequences include a loss of self-confidence and social isolation, along with other negative outcomes such as anxiety, depression, deterioration of sex life, and a decrease in physical activity. All of these conditions are associated with a poor quality of life (QoL), a generic term that describes the quality of life in many areas of human life and reflects individual or societal expectations regarding well-being [8]. UI is a significant health problem with physical, social, economic, and psychological consequences on patients and their quality of life [9].

According to the World Health Organization (WHO), quality of life is the subjective perception of one’s own life situation, evaluated within the cultural context and value systems in which we live and in relation to our own goals, expectations, norms, and concerns [10].

The impact of UI on quality of life is evident, and it is often the psychological and social consequences that strongly motivate patients to seek treatment. Therefore, the International Continence Society (ICS) recommends incorporating quality-of-life assessment measures into clinical practice to improve patients’ perception of their health status. These assessment questionnaires have become common in recent decades with the emergence of generic and specific instruments for certain pathologies [2]. Several questionnaires for evaluating the quality of life regarding UI are used internationally after appropriate official linguistic validation. Typical questionnaires include the I-QoL and the King’s Health Questionnaire [11]. 

Despite an increasing number of medical studies indicating the importance of quality of life and the high prevalence of urinary incontinence, no study in our Moroccan context has assessed the quality of life in patients suffering from urinary incontinence. Hence, the objective of our study is to determine the impact of urinary incontinence on quality of life and to identify the determinants of this quality of life in patients with this condition.

## Materials and methods

Study design and population

This is a cross-sectional study conducted at the diagnostic center of CHU Hassan II in Fez, Morocco, between June and September 2019. Included in the study were female and male patients over 18 years of age who consulted the diagnostic center of CHU Hassan II de Fez for any health issue, and we asked them about the presence of urinary incontinence. We then included only those patients who reported suffering from urinary incontinence. Exclusion criteria comprised being pregnant at the time of the survey, having a urinary tract infection in the previous month, experiencing challenges in responding to the questionnaire, and refusing to participate.

Data collection

The questionnaire included sociodemographic data (gender, age, marital status, level of education, occupation, place of residence, type of health insurance); patient history (number of pregnancies, number of vaginal deliveries, number of cesarean sections, number of episiotomies, number of perineal rehabilitation sessions, number of instrumental deliveries and menopause); characteristics of UI (type, frequency, importance, duration, severity, number of medical consultations in the last year, and history of surgery for UI). We also administered the Moroccan Arabic dialect version of the I-QoL (incontinence quality of life) questionnaire [12] to assess the quality of life in patients suffering from UI.

We defined UI as any involuntary leakage of urine present at the time of the study, regardless of its characteristics in terms of duration, frequency, or quantity [1]. We classified patients into three types: stress UI, urgency UI, and mixed UI. We assessed the severity of UI by multiplying the results of questions about the frequency and quantity of urine lost, according to the Sandvik formula [13]. This score defined three levels of severity: 1-2 = mild UI; 3-4 = moderate UI; and 5-6 = severe UI.

Incontinence quality of life (I-QoL) questionnaire

The I-QoL (incontinence quality of life) questionnaire, which we used in the present study, was recommended as the preferred method at the second International Consultation on Incontinence for assessing the quality of life in patients with UI [14].

The I-QoL was developed by Wagner [15] and Patrick [16]. It is a self-assessment questionnaire commonly used for individuals suffering from UI. The I-QoL consists of 22 items, which are grouped into three subscales: avoidance and limiting behaviors (ALB), psychosocial impact (PSI), and social embarrassment (SE), all based on a five-point Likert scale, where 1 = always, 2 = often, 3 = sometimes, 4 = rarely, and 5 = never.

The total I-QoL score, as well as the score for each subscale, is calculated by summing the response for each item. The lowest possible score is then subtracted, and the result is divided by the possible raw score. The results are then transformed to obtain a range from 0 (poor quality of life) to 100 (best quality of life). This instrument has been widely used and successfully validated for individuals with UI [17]. In our study, we used the version translated and validated in Moroccan Arabic language [12].

The formula used to calculate the score is as follows:

Total score = ([each item as a whole - the lowest possible score] / the possible raw score) * 100

Data analysis

We excluded questionnaires with missing data. We conducted data entry at the Laboratory of Epidemiology, Clinical Research, and Community Health of the Faculty of Medicine, Pharmacy, and Dentistry in Fez, Morocco. We entered and processed the data in Excel 2010 (Redmond, USA).

We used descriptive statistics to describe sociodemographic data, clinical data, and quality-of-life scores. We presented quantitative variables as means and standard deviations (SD) and qualitative variables as percentages. We employed univariate analyses to determine factors associated with quality of life, using analysis of variance (ANOVA) for qualitative variables (comparison of multiple averages) and simple linear regression for quantitative variables (comparison of two quantitative averages), and we used the student t-test for comparison of two means.

Subsequently, we conducted a multivariate analysis using stepwise multiple linear regressions to identify factors associated with the total I-QoL score and its subscales. We selected independent variables for the model based on univariate analysis with a threshold p-value of 0.20. We performed statistical analyses using IBM Corp. Released 2019. IBM SPSS Statistics for Windows, Version 26.0. Armonk, NY: IBM Corp. and defined statistical significance as p < 0.05.

Ethical considerations

We obtained approval from the ethics committee at the University Hospital Center Hassan II of Fez and consent from the Ministry of Health. We obtained the free and informed consent of participants before filling out the questionnaires. Additionally, the collected information was entirely confidential and used only for research purposes. A trained investigator from the research group conducted an interview during the urological consultation to fill out a questionnaire. The interview took place in accordance with medical ethics, in a closed, quiet room, with only the participant and the investigator present to respect their privacy.

## Results

Sociodemographic characteristics

The sociodemographic characteristics of the patients are shown in Table 1. A total of 278 patients with UI participated in the study. About 78% were women, 21.2% were men, and 70.9% were over 50 years old. The majority of the patients were married (74.8%), and more than half of our sample were housewives (56.5%). In addition, 75.9% lived in urban areas, 34.1% were illiterate, and 60.1% were covered by RAMED. The univariate analysis revealed several factors associated with a better quality of life (high total I-QoL score). including young age (p = 0.002), single marital status (p = 0.002), higher education level (p < 0.001), urban residence (p < 0.001), and being a student (p = 0.001) (Table 1).

**Table 1:** Quality of life-associated factors (sociodemographic factors) in bivariate analysis using t-tests and ANOVA analysis (statistical significance was defined as p < 0.05) ALB: Avoidance and limiting behaviors, PSI: Psychosocial impact, SE: Social embarrassment, SD: Standard deviation

Variable	Number/%	Total score means ± SD	p	ALB means ± SD	p	PSI means ±SD	p	SE means ± SD	p
Age (t test)			0.002		0.016		0.002		0.001
20–50	80/29.1%	53.02 ±24.48		46.91 ±24.96		61.21 ±25.55		48.06 ±28.92	
51–95	195/70.9%	42.93 ±24.34		39.21 ±23.39		50.64 ±26.03		35.03 ±28.783	
Gender (t test)			0.77		1		0.483		0.936
Men	59/21.2%	45.26 ±23.72		41.52 ±23.69		51.88 ±26.71		39.32 ±26.17	
Women	219/78.8%	46.30 ±25.13		41.52 ±24.21		54.60 ±26.33		38.97 ±30.39	
Marital status (ANOVA)			0.002		0.002		0.007		0.005
Married	208/74.8%	45.86 ±24.713		41.24 ±23.94		53.76 ±26.62		39.04 ±29.36	
Single	18/6.5%	65.66 ±16.685		60.59 ±16.54		73.14 ±16.80		60.28 ±24.93	
Divorced	15/5.4%	42.35 ±17.13		39.58 ±14.64		51.11 ±19.58		31.00 ±21.89	
Widower	37/13.3%	39.25 ±27.04		34.62 ±26.70		47.37 ±27.71		32.03 ±30.92	
Study level (ANOVA)			<0.001		<0.001		<0.001		<0.001
Illiterate	94/34.1%	39.71 ±23.54		36.10 ±22.52		46.95 ±24.76		32.45 ±29.34	
Koranic	39/14.1%	36.95 ±23.14		33.65 ±23.79		44.37 ±23.72		28.85 ±26.04	
Primary	57/20.7%	48.09 ±26.33		41.99 ±24.89		57.89 ±28.46		40.18 ±30.26	
Secondary	52/18.8%	53.56 ±24.43		48.49 ±23.60		61.11 ±26.63		48.08 ±29.50	
Superior	34/12.3%	57.12 ±19.12		52.20 ±20.79		65.27 ±20.90		50.29 ±23.05	
Profession (ANOVA)			0.001		<0.001		0.003		0.002
Student	4/1.4%	69.60 ±16.65		68.75 ±22.67		77.77 ±10.63		56.25 ±21.36	
Housewife	156/56.5%	43.26 ±25.02		38.24 ±23.67		52.10 ±26.58		35.38 ±30.36	
Not employed	56/20.3%	40.69 ±22.31		37.50 ±21.45		47.02 ±25.44		34.37 ±26.76	
Part-time work	29/10.5%	52.23 ±24.11		47.30 ±23.26		59.86 ±25.93		46.38 ±26.98	
Full-time work	31/11.2%	59.20 ±22.71		54.13 ±24.19		65.86 ±22.91		55.32 ±26.80	
Residence (t-test)			<0.001		<0.001		<0.001		<0.001
Rural	66/24.1%	34.16 ±23.27		30.30 ±23.17		42.12 ±24.15		25.98 ±27.73	
Urban	208/75.9%	49.96 ±24.01		45.14 ±23.08		57.97 ±25.95		43.22 ±28.87	

History of patients with UI

Table 2 summarizes the history of the patients who participated in our study. The average number of pregnancies among women was 4.30 (± 2.709), with an average of deliveries through vaginal or cesarean section being 3.92 (± 2.735) and 0.24 (± 0.553), respectively. Simple linear regression analysis showed that when the average number of pregnancies and vaginal deliveries increased by one unit, the quality-of-life score decreased by -2.478 β (95% CI -3.684; -1.272) and -2.382 β (95% CI -3.586; -1.179) on average, respectively.

**Table 2:** Factors associated with quality of life (historical factors) in bivariate analysis using simple linear regression ALB: Avoidance and limiting behaviors, PSI: Psychosocial impact, SE: Social embarrassment, SD: Standard deviation

Simple linear regression		TOTAL SCORE		ALB		PSI		SE	
Variable	Means ±SD	β	95%CI	β	95%CI	β	95%CI	β	95%CI
Number of Pregnancy	4.30 ± 2.709	-2.478	-3.684;-1.272	-2.833	-3.970; -1.697	-2.000	-3.288; -0.711	-2.770	-4.241 ; -1.298
Number of Vaginal Deliveries	3.92 ± 2.735	-2.382	-3.586;-1.179	-2.75	-3.884; -1.617	-1.921	-3.205; -0.637	-2.624	-4.092; -1.156
Number of C-sections	0.24 ± 0.553	4.299	-1.884; 10.483	4.169	-1.755; 10.092	4.343	-2.165; 10.851	4.431	-3.073; 11.934
Number of Episiotomies	0.75 ± 1.296	-0.779	-3.485; 1.927	-1.119	-3.703; 1.465	-0.381	-3.225; 2.463	-0.951	-4.245; 2.343
Number of Perineal Rehabilitations	0.05 ± 0.266	-9.758	-22.885; 3.369	-5.608	-18.266; 7.051	-10.893	-24.554; 2.768	-14.355	-30.540; 1.830
Number of Instrumental Deliveries	0.18 ± 0.479	-0.396	-7.542; 6.749	-0.949	-7.795; 5.897	-1.086	-8.595; 6.423	1.729	-7.106; 10.565

Clinical characteristics of UI

The various clinical characteristics of our study sample are grouped in the following table (Table 3). About half (48.2%) of our study population were overweight, and more than half of the women were menopausal (62.8%). Urgency UI was the most common form in our sample, accounting for 38.6% of cases. Approximately 90% of patients experienced leaks in small quantities, and 50.9% reported having between five and 10 urinations per day. 

**Table 3:** Quality-of-life-associated factors in bivariate analysis (clinical factors) using t-test and ANOVA analysis (statistical significance was defined as p < 0.05) ALB: Avoidance and limiting behaviors, PSI: Psychosocial impact, SE: Social embarrassment, SD: Standard deviation, UI: Urinary incontinence

Variable	Number /%	Total score Mean ±SD	p	ALB Mean ±SD	p	PSI Mean ±SD	p	SE Mean ±SD	p
Menopause (t test)			<0.001		0.001		0.001		<0.001
Yes	130/62.8%	40.81 ±23.79		36.53 ±21.98		49.72 ±25.65		31.62 ±28.67	
No	77/37.2%	53.79 ±24.27		47.68 ±24.89		61.86 ±25.25		49.03 ±29.36	
Type of UI (ANOVA)			<0.001		<0.001		<0.001		<0.001
Stress	73/26.8%	49.28 ±21.69		45.54 ±20.21		57.03 ±23.51		54.32 ±33.56	
Urgency	105/38.6%	53.16 ±24.05		49.28 ±22.94		60.23 ±25.69		59.95 ±34.72	
Mixed	94/34.6%	35.03 ±23.39		28.82 ±21.84		44.53 ±25.86		37.50 ±31.10	
Leak frequency (ANOVA)			<0.001		<0.001		<0.001		<0.001
Less than once a month	53/19.1%	64.47 ±21.38		61.02 ±21.63		71.59 ±20.59		57.17 ±27.89	
Once or more per month	95/34.2%	51.79 ±20.82		48.65 ±18.39		58.97 ±22.61		43.89 ±27.56	
Once or more per week	87/31.3%	35.59 ±22.88		30.63 ±21.77		43.99 ±25.03		28.39 ±27.60	
Every day and/or night	43/15.5%	31.98 ±22.73		23.76 ±18.61		41.73 ±28.85		27.56 ±26.05	
Importance of leaks (ANOVA)			<0.001		<0.001		<0.001		<0.001
A few drops	122/43.9%	55.38 ±24.58		49.15 ±24.04		62.75 ±26.17		52.09 ±29.13	
A small volume	127/45.7%	40.20 ±21.50		37.08 ±21.22		48.57 ±23.17		30.12 ±25.10	
A big volume	29/10.4%	32.64 ±25.93		28.87 ±26.31		41.18 ±29.61		23.28 ±27.29	
Severity of UI (ANOVA)			<0.001		<0.001		<0.001		<0.001
Lighter	97/34.9%	62.55 ±20.50		57.79 ±19.87		69.78 ±21.12		57.11 ±26.66	
Moderate	84/30.2%	41.83 ±22.79		37.83 ±21.44		49.07 ±25.05		35.18 ±27.93	
Severe	97/34.9%	33.28 ±21.22		28.44 ±20.62		42.55 ±24.80		24.33 ±23.75	
Duration of UI (ANOVA)			<0.001		<0.001		<0.001		0.001
<1 month	15/5.4%	62.20 ±26.75		61.04 ±24.88		66.85 ±30.16		55.67 ±28.27	
1–6 month	27/9.7%	43.35 ±32.25		39.46 ±32.66		50.41 ±32.42		36.85 ±35.33	
6 month-1 year	45/16.2%	53.23 ±23.68		47.63 ±22.99		62.22 ±24.01		46.00 ±29.28	
1–5 years	101/36.5%	49.65 ±23.23		43.87 ±22.46		58.30 ±25.17		43.32 ±28.44	
5–10 years	58/20.9%	36.97 ±20.86		34.32 ±20.95		43.77 ±23.34		28.97 ±25.23	
>10 years	31/11.2%	35.85 ±21.66		30.84 ±18.76		44.62 ±23.40		28.06 ±28.59	
Frequency of daily urination (ANOVA)			<0.001		<0.001		<0.001		<0.001
1–4	92/33.2%	55.15 ±24.77		50.44 ±23.92		63.19 ±25.71		48.21 ±31.53	
5–10	141/50.9%	43.28 ±24.11		38.34 ±23.37		51.43 ±25.94		36.49 ±28.16	
>10	44/15.9%	36.73 ±21.45		33.52 ±21.47		43.87 ±23.79		28.98 ±24.10	
Wearing protection (t test)			<0.001		<0.001		<0.001		<0.001
Yes	127/46%	36.62 ±24.04		32.30 ±22.74		45.31 ±26.19		27.87 ±27.84	
No	149/54%	53.84 ±22.69		49.07 ±22.45		61.26 ±24.47		48.12 ±27.60	
Frequent need to change underwear (t test)			<0.001		<0.001		<0.001		<0.001
Yes	202/73.2%	37.60 ±21.41		33.92 ±21.19		45.51 ±23.29		29.23 ±25.08	
No	74 /26.8%	68.63 ±18.52		61.65 ±19.27		76.87 ±20.34		64.93 ±24.27	
Number of medical consultations (ANOVA)			0.017		0.028		0.009		0.073
0	63/22.7%	46.95 ±19.68		40.77 ±20.27		55.33 ±21.43		41.75 ±26.26	
1–3	139/50.2%	47.40 ±25.96		42.64 ±24.85		56.17 ±27.09		39.21 ±30.75	
4–7	65/23.5%	45.63 ±26.53		42.59 ±25.58		52 ±28.74		39.00 ±30.13	
7–11	10/3.6%	21.82 ±11.01		19.37 ±8.56		27.50 ±14.84		15.50 ±13.42	

Regarding the duration of symptoms, over half (68.6%) had been suffering from UI for more than a year, 46% used protective measures such as sanitary pads and diapers, and the majority reported the frequent need to change underwear (73.2%). About 35% suffered from severe UI, and nearly half (50.2%) reported consulting a doctor one to three times a year.

The univariate analysis showed that all of the following were associated with a better quality of life: urgency UI (p < 0.001), non-menopausal women (p < 0.001), those reporting leaks less than once a month (p < 0.001), those with leaks in small quantities (p < 0.001), lighter urinary incontinence (p < 0.001), symptoms lasting less than one month (p < 0.001), and a daily frequency of urination of one to four times per day (p < 0.001). Additionally, not needing to change underwear frequently (p < 0.001) or using protection (p < 0.001) improved our patient's quality of life. Our analysis further demonstrated a significant association between the severity of UI and protective behaviors: 83.5% of patients with moderate and severe UI used a protection measure (p < 0.001), and 73.8% changed underwear frequently (p < 0.001).

Subscales and total score I-QoL

The scores for the different scales ranged from 39.05 to 54.02. The social embarrassment scale obtained the lowest score, 39.05 (± 29.50); the psychosocial impact scale had a mean score of 54.02 (± 26.39), and the avoidance and limiting behaviors had a mean score of 41.52 (± 24.05). The total I-QoL score was 46.08 (± 24.80).

Multivariate analysis

The results of the multiple linear regression model are presented in Table 4.

**Table 4:** Multivariate (stepwise) analysis of quality-of-life predictors adjusted R² using multiple linear regression. ALB: Avoidance and limiting behaviors, PSI: Psychosocial impact, SE: Social embarrassment, SD: Standard deviation, UI: Urinary incontinence

VARIABLE (Multiple linear regression)	Total score (R²=0.50)		ALB (R²=0.48)		PSI (R²=0.43)		SE (R²=0.46)		
	β	95%CI	β	95%CI	β	95%CI	β	95%CI	
Residence									
Rural	-8.094	-13.043; -3.145	-6.90	-11.733; -2.067	-8.719	-14.386; -3.051	-8.882	-14.971;-2.794	
Urban									
Severity of UI									
Lighter	11.211	1.748; 20.674	1.796	-7.445; 11.038	10.907	0.071; 21.743	26.822	15.181; 38.464	
Moderate	3.846	-2.743; 10.435	-0.60	-7.036; 5.835	2.468	-5.078; 10.013	13.441	5.334; 21.548	
Severe	-	-	-	-	-	-	-	-	
Frequency of daily urination									
1–4	8.062	1.471; 14.653	5.695	-0.741; 12.132	9.733	2.185;17.28	8.843	0.734; 16.951	
5–10	2.294	-3.727; 8.314	0.013	-5.867; 5.892	4.036	-2.858; 10.93	2.808	-4.599; 10.215	
>10	-	-	-	-	-	-	-	-	
Wearing protection									
No	9.466	4.987; 13.946	8.184	3.809; 12.559	8.75	3.620; 13.880	12.807	7.296; 18.319	
Yes	-	-	-	-	-	-	-	-	
Frequent need to change underwear									
No	24.871	19.975; 29.767	20.984	16.203; 25.766	25.694	20.087;31.3	29.608	23.585; 35.631	
Yes									

Total score I-QoL

The multivariate analysis using stepwise multiple linear regression revealed that the following factors were significantly associated with a better quality of life-related to UI (high total I-QoL score): lighter UI (β = 11.211; 95% CI: 1.748; 20.674); daily frequency of urination between one to four per day (β = 8.062; 95% CI: 1.471; 14.653); not needing to use protection (β = 9.466; 95% CI: 4.987; 13.946); or frequently changing underwear (β = 24.871; 95% CI: 19.975; 29.767).

On the other hand, a factor significantly associated with a lower quality of life related to UI (low total I-QoL score) was rural residence (β = -8.094; 95% CI: -13.043; -3.145) after adjusting for all variables.

Avoidance and limiting behavior subscale (ALB)

The multivariate analysis revealed that factors significantly associated with a better quality of life were not needing to use protection (β = 8.184; 95% CI: 3.809; 12.559) and frequently changing underwear (β = 20.984; 95% CI: 16.203; 25.766). A factor significantly associated with a lower quality of life was rural residence (β = -6.900; 95% CI: -11.733; -2.067) after adjusting for all variables.

Psychosocial impact subscale (PSI)

The multivariate analysis revealed that factors significantly associated with a better quality of life were lighter UI (β = 10.907; 95% CI: 0.071; 21.743); frequency of urination between one and four times per day (β = 9.733; 95% CI: 2.185; 17.280); not needing to use protection (β = 8.750; 95% CI: 3.620; 13.880); and frequently changing underwear (β = 25.694; 95% CI: 20.087; 31.300).

Meanwhile, a factor significantly associated with a lower quality of life related to UI was rural residence (β = -8.719; 95% CI: -14.386; -3.051) after adjusting for all variables.

Social embarrassment subscale (SE)

The multivariate analysis revealed that factors significantly associated with a better quality of life were as follows: lighter UI (β =26.822; 95% CI: 15.181; 38.464) and moderate UI (β =13.441; 95% CI: 5.334; 21.548), frequency of urination between one and four times per day (β =8.843; 95% CI: 0.734; 16.951); not needing to use protection (β =12.807; 95% CI: 7.296; 18.319); and frequently changing underwear (β = 29.608; 95% CI: 23.585; 35.631).

Meanwhile, a factor significantly associated with a lower quality of life related to UI was rural residence (β = -8.882; 95% CI: -14.971; -2.794) after adjusting for all variables.

## Discussion

In order to explore the relevant components of quality of life related to UI, our study aims to assess the quality of life of patients suffering from this disease in Morocco.

Our participants reported an average total I-QoL score of 46.08 ± 24.80, as well as average scores for the subscales: avoidance and limiting behavior, psychosocial impact, and social embarrassment, respectively, of 41.52 ± 24.05, 54.02 ± 26.39, and 39.05 ± 29.50.

The overall I-QoL scores suggest that UI has the greatest impact on social embarrassment.

In a study conducted by Hwa Su Lim et al. [18], they found a slightly higher quality of life related to UI (total I-QoL score of 61.1 ± 21.0) and a mean score for the subscales practically closer to our results: ALB (45.9 ± 23.4), PSI (48.4 ± 26.2), and SE (34.9 ± 26.5).

A study conducted by Magdaléna Hagovska et al. in the Slovak Republic reported a higher quality-of-life score related to UI (total I-QoL score of 82.9 ± 13.8). The scores for the subscales for avoidance and limiting behavior, psychosocial impact, and social embarrassment were 80.8 ± 14.6, 84.9 ± 14.5, and 82.7 ± 14.5, respectively. This difference may be attributed to the participants’ engagement in physical activities [19].

Sotiria Papanicolaou et al. [20] also reported a better quality of life related to UI, with an average total I-QoL score of 80.2 and average scores for the subscales: avoidance and limiting behavior (74.9), psychosocial impact (89.6), and social embarrassment (71.9).

According to the results of the multivariate analysis. There is a correlation between the severity of UI and quality of life. Lighter UI is associated with a better quality of life compared to moderate and severe UI. However, we found no correlation between the avoidance and limiting behavior subscale and the severity of UI. This may be explained by the fact that regardless of the severity of UI, incontinent patients tend to have a limiting behavior.

Our results are consistent with previous studies that also revealed significant correlations between the total I-QoL score and the severity of UI. The findings suggest that increasing severity of UI has a negative impact on the quality of life [20]. Hwa Su Lim et al. found that patients who experienced significant urinary leakage before surgery had lower total I-QoL scores compared to patients with smaller amounts of urinary leakage [18], which is entirely consistent with the literature in that people suffering from severe UI report a greater impairment of quality of life [21]. The same result was reported by Francesca Chiaffarinoa et al. [22] in a study on the impact of urinary incontinence and overactive bladder on quality of life, based on the SF-12 questionnaire validated for Italian women.

The frequency of urinary incontinence has been variably assessed in the literature. It has been observed that less frequent urinary leaks have a positive influence on quality of life. Despite the difficulties of comparing data found in the literature due to methodological differences and the wide variety of questionnaires used to assess UI-related quality of life, an Arab study showed that women who lost urine more often and in greater quantity sustained a greater impact on quality of life, achieving the worst questionnaire scores compared with women suffering from less frequent leakage [23]. It should be noted that some studies showed that the severity of symptoms, measured by the quantity and frequency of urine loss, is the most important predictor of poorer quality of life [21,24].

In a study involving 1,203 women with UI in four European countries, those with higher volumes of urine loss reported a greater frequency of daily incontinence episodes, as well as a more detrimental impact on all aspects of quality of life as assessed by the Health-Related Quality of Life (HRQOL) questionnaire. At the same time, these women also reported a more marked deterioration in mental well-being [24]. However, in a study involving 39 patients, a reduction in the severity of leaks was not always sufficient to improve the quality of life; only complete healing mattered [25]. The majority of studies have concluded that the impact of UI on quality of life will vary depending on the severity of the condition as well as the patients’ activities and interests [26]. People affected by UI develop coping strategies to deal with uncomfortable situations, such as the use of protective measures, limiting their participation in activities, and avoiding extended walks, trips, and activities involving close social contact. Overall, these behaviors contribute to a general deterioration in their quality of life. Unfortunately, however, few studies have examined these adaptation strategies [21].

In our study, the increased need for sanitary protection and frequent underwear changes were associated with a deterioration of quality of life. Our results concur with those of Paul Abrams et al. [24], who found that quality of life decreased with the severity of UI symptoms, and women with severe UI were more likely to use sanitary protection specifically designed for UI and to change underwear frequently because of wetness and fear of being embarrassed.

Finally, the place of residence is an important determinant; people living in rural areas have a lower quality of life compared to those living in urban areas. The interest in this variable stems from the inequalities observed between rural and urban areas. In general, there is a high concentration of healthcare infrastructures in urban areas, to the detriment of rural areas. People living in urban areas have easier access to health care (Haddad et al., 2004) [27]. The difference in utilization between rural and urban areas is linked to several factors such as poverty, population level of education, receptiveness to modern medicine, geographical and financial accessibility of modern health services, and adherence to medical insurance [28].

A limitation of our study is that the sample was collected only from a large central hospital. However, we included objective measures of UI, by which participants were classified into three groups (lighter UI, moderate UI, and severe UI) according to the degree of severity, providing further evidence of the relationship between the severity of the condition and quality of life.

Nevertheless, future studies should examine how individuals cope with UI over time, with a particular focus on the role of managing the relationship between psychological morbidity, suffering, and quality of life. Also, future research should assess social support and body image as mediators in the relationships between sexual satisfaction and quality of life, especially in larger samples of individuals suffering from UI.

## Conclusions

Our study revealed that several factors influenced the quality of life related to UI, particularly the severity of UI incontinence and the frequency of leaks. The assessment of quality of life should be considered as a central parameter in the evaluation of UI, on the basis of which the actions of healthcare professionals will focus during the planning and guidance of treatment, which are essential to the healing process. As a result, these professionals must prioritize assistance focused on health promotion and therapeutic processes, primarily seeking to improve the quality of life for patients.
